# Congenital rubella syndrome surveillance in India, 2016–21: Analysis of five years surveillance data

**DOI:** 10.1016/j.heliyon.2023.e15965

**Published:** 2023-05-09

**Authors:** Devika Shanmugasundaram, Sanjay Verma, Kuldeep Singh, Bhagirathi Dwibedi, Shally Awasthi, S. Mahantesh, Himabindu Singh, Sridhar Santhanam, Nivedita Mondal, Geetha S, Priya Sreenivasan, Shikha Malik, Manish Jain, Rajlakshmi Viswanathan, Shalini Tripathi, Bhupeshwari Patel, Gajanan Sapkal, R. Sabarinathan, Mini P. Singh, R.K. Ratho, Vijaylakshmi Nag, Ravishekhar Gadepalli, Tapas Kumar Som, Baijayantimala Mishra, Amita Jain, M. Ashok, Devara Sudha Madhuri, V Sudha Rani, Asha Mary Abraham, Deepa John, Rahul Dhodapkar, A. Syed Ali, Debasis Biswas, Deepashri Pratyeke, Ashish Bavdekar, Jayant Prakash, Varsha Singh, Nidhi Prasad, Jaydeb Ray, Agniva Majumdar, Shanta Dutta, Nivedita Gupta, Manoj Murhekar, CRS Surveillance Group, Akhil Sharma, Aniruddha Ghosh, Arun Alexander, Arun Baranwal, Avinash Anantharaj, Adhisivam Bethou, Dolat S. Shekhawat, G. Kiruthika, Jagat Ram, Madhu Gupta, Mamatha Gowda, Manoj K Rohit, Nabaneeta Dash, Naveen Sankhyan, Nidhi Kaushal, Niranjan Hunasanahalli Shivanna, Nirupama Kasturi, P. Prem Kumar, Parul Chawla Gupta, Pradeep Kumar Gunasekaran, Pratibha Singh, Praveen Kumar, Sanjay Kumar Munjal, Siddharth Agarwal, Suhani Manasa, Suruchi Shukla, Urvashi Nehra, Valsan P Verghese, Varuna Vyas, Vikas Gupta

**Affiliations:** a ICMR–National Institute of Epidemiology, Chennai, India; b Postgraduate Institute of Medical Education and Research, Chandigarh, India; c All India Institute of Medical Sciences, Jodhpur, India; d All India Institute of Medical Sciences, Bhubaneswar, India; e King George's Medical University, Lucknow, India; f Indira Gandhi Institute of Child Health, Bengaluru, India; g Niloufer Hospital, Hyderabad, India; h Christian Medical College, Vellore, India; i Jawaharlal Institute of Postgraduate Medical Education and Research, Pondicherry, India; j Government Medical College, Trivandrum, India; k All India Institute of Medical Sciences, Bhopal, India; l Mahatma Gandhi Institute of Medical Sciences, Sewagram, India; m ICMR-National Institute of Virology, Pune, India; n KEM Hospital, Pune, India; o Indira Gandhi Institute of Medical Sciences, Patna, India; p Institute of Child Health, Kolkata, India; q ICMR-National Institute of Cholera and Enteric Diseases, Kolkata, India; r Indian Council of Medical Research, New Delhi, India

**Keywords:** Congenital Rubella Syndrome, Surveillance, India

## Abstract

**Background:**

In India, facility-based surveillance for congenital rubella syndrome (CRS) was initiated in 2016 to estimate the burden and monitor the progress made in rubella control. We analyzed the surveillance data for 2016–2021 from 14 sentinel sites to describe the epidemiology of CRS.

**Method:**

We analyzed the surveillance data to describe the distribution of suspected and laboratory confirmed CRS patients by time, place and person characteristics. We compared clinical signs of laboratory confirmed CRS and discarded case-patients to find independent predictors of CRS using logistic regression analysis and developed a risk prediction model.

**Results:**

During 2016–21, surveillance sites enrolled 3940 suspected CRS case-patients (Age 3.5 months, SD: 3.5). About one-fifth (n = 813, 20.6%) were enrolled during newborn examination. Of the suspected CRS patients, 493 (12.5%) had laboratory evidence of rubella infection. The proportion of laboratory confirmed CRS cases declined from 26% in 2017 to 8.7% in 2021. Laboratory confirmed patients had higher odds of having hearing impairment (Odds ratio [OR] = 9.5, 95% confidence interval [CI]: 5.6–16.2), cataract (OR = 7.8, 95% CI: 5.4–11.2), pigmentary retinopathy (OR = 6.7, 95 CI: 3.3–13.6), structural heart defect with hearing impairment (OR = 3.8, 95% CI: 1.2–12.2) and glaucoma (OR = 3.1, 95% CI: 1.2–8.1). Nomogram, along with a web version, was developed.

**Conclusions:**

Rubella continues to be a significant public health issue in India. The declining trend of test positivity among suspected CRS case-patients needs to be monitored through continued surveillance in these sentinel sites.

## 1 Introduction

Rubella is one of the commonest causes of febrile rash among children in India. Rubella infection in the first trimester of pregnancy can lead to a variety of adverse outcomes including spontaneous abortion, still birth or birth of a baby with several birth defects. This is referred to as congenital rubella syndrome (CRS) [1,2]. India along with other countries in the WHO-Southeast Asia Region have resolved to eliminate measles and Rubella/CRS by 2023 [3]. The key strategies to achieve the elimination goals include (a) achieving and maintaining high population immunity by ensuring high (>95%) coverage of two doses of measles and rubella containing vaccine (MRCV) in every district (b) developing and sustaining a sensitive case-based surveillance for measles and rubella/CRS (c) developing and maintaining proficient measles and rubella laboratory network and (d) ensuring adequate outbreak preparedness and rapidly responding to measles and rubella outbreaks [4]. Between 2017 and 2019, India conducted supplementary immunization activity (SIA) with MRCV, for children aged 9 months and <15 years in order to increase population immunity. These campaigns have been completed in 34 of the 36 Indian States, vaccinating approximately 324 million children with MRCV [[5], [6], [7]]. A facility-based surveillance was initiated in five sentinel hospitals [8,9] in November 2016, which was expanded to additional seven sites in March 2019 and additional two sites in April 2020 (Supplementary figure 1). The objective of CRS surveillance was to estimate the disease burden and monitor the progress made in rubella control.

We analyzed the surveillance data for 2016–2021 to describe the epidemiology of CRS in India. We assessed the quality of CRS surveillance against the indicators recommended by the World Health Organization (WHO) [10]. Using the surveillance data, we also constructed a risk model to identify predictors of CRS and developed a nomogram to calculate probability of CRS in an infant, based on clinical signs.

## 2 Methods

### 2.1 Case finding and data collection

The CRS surveillance aimed at identifying infants (age: 0–11 months) with suspected CRS either during the examination of newborns born at the sentinel sites, or among children attending out-patient departments (OPDs) of these hospitals. Infants presenting with any of the following conditions were suspected of CRS: (a) structural heart defect confirmed by echocardiography (b) hearing impairment confirmed by brainstem evoked response audiometry (BERA), or auditory steady-state response (ASSR) or two ‘refer’ otoacoustic emission (OAE) tests (c) presence of one or more of the eye signs: cataract, microphthalmos, micro-cornea, congenital glaucoma and pigmentary retinopathy (d) maternal history of suspected rubella infection during pregnancy and (e) Strong clinical suspicion of CRS (8,9). Such infants were enrolled in the surveillance after obtaining written informed consent from their parents. Using a standard case-report form, information about demography, and clinical details of the suspected CRS patients were collected. All suspected CRS patients underwent evaluation for heart defect, eye examination and hearing assessment.

### 2.2 Laboratory investigation

From every suspected CRS case-patient, 1–2 ml of blood was collected, and serum samples were tested for the presence of IgM antibodies against rubella using a commercial Enzyme Linked Immunosorbent Assay (ELISA) kit (Euroimmun, Luebeck, Germany) as per manufacturer’s instructions. Serum samples from children aged 6–11 months were also tested for IgG antibodies against rubella using commercial ELISA kit (Euroimmun, Luebeck, Germany). Additional blood samples were collected from children aged <1 month with negative rubella IgM serology, and infants aged 6–11 months if they had negative rubella IgM but positive rubella IgG serology. Oro-pharyngeal (OP) swabs were collected from all the laboratory confirmed rubella cases and tested for rubella virus by reverse transcriptase polymerase chain reaction (RT-PCR). Laboratory confirmed CRS case-patients were followed up (either by requesting the parents to come to the sentinel hospitals or telephonically if the parents were unable to come to the hospital) for viral excretion.

### 2.3 Case definitions

Based on the rubella serology results and clinical signs, suspected CRS patients were categorized into one of the following categories: (a) Clinically compatible case: Infants having two signs from group A (Congenital cataract, congenital glaucoma, pigmentary retinopathy, congenital heart defect, or hearing impairment) or one sign from group A and one sign from group B (microcephaly, developmental delay, meningoencephalitis, splenomegaly, purpura, radiolucent bone disease, or jaundice with onset within 24 h after birth), in the absence of rubella serological results (b) Laboratory confirmed CRS: presence of at least one group A sign, along with serological evidence of rubella infection (presence of IgM antibodies or sustained level of IgG antibodies, as determined on at least two specimens collected at least 1 month apart) (c) Congenital rubella infection (CRI): Serological evidence of rubella infection among infants without any signs from group A and (d) Discarded case: negative rubella serology irrespective of clinical signs [4,10].

### 2.4 Quality of surveillance data

For assessing the quality of CRS surveillance, WHO has proposed eight indicators related to reporting, adequacy of investigation in terms of key data points, specimen collection and testing, viral detection, virus excretion, timeliness of detection of confirmed CRS cases after birth, specimen transport and testing [11,12].

### 2.5 Data analysis

We analyzed the surveillance data for the period 2016–2021 to describe the distribution of suspected and laboratory confirmed CRS patients by person (age, sex and clinical details), place (district) and time. As the new surveillance sites were added in different years and a few sites discontinued in between, we calculated rubella positivity for each site by year and used χ^2^ test for trend to find out if there was a linear trend. We summarized the categorical variables with percentages, and continuous variables with means (standard deviation (SD)) or median (interquartile range (IQR)). χ^2^ test for trend was calculated for sites having at least three years of data.

We compared group A and group B signs along with demographic characteristics (age and sex) of the laboratory confirmed CRS and discarded case-patients to find independent predictors of CRS using univariate binary logistic regression. Clinically compatible cases and congenital rubella infections were excluded from the analysis. Patients who underwent complete clinical examination for hearing impairment, eye signs, congenital heart defects, general and CNS examinations were included in the regression model. -2log likelihood criterion was used to select the best model for inclusion in multivariable binary logistic regression. The receiver operating characteristics (ROC) curve was plotted to evaluate the performance of the predictive model and C-index was expressed by the area under ROC curve (AU-ROC) which was used to assess the discriminative power of the predictive model that classifies infants into confirmed CRS or not. A nomogram was developed based on the final predictive model [13].

For validation of the developed model, bootstrapping technique was performed with 1000 re-samples. AU-ROC curve was calculated for original and bootstrap data to assess the discriminative power of the developed model. We calculated apparent performance (performance of model on entire original data), bootstrap performance (performance of model on bootstrap sample) and test performance (performance of bootstrap model on the original data) in terms of AU-ROC. Optimism was calculated as the average difference between the bootstrap performance and the test performance over 1000 re-samples. Optimism value was subtracted from the apparent performance to obtain bias-corrected estimate of the performance (validated performance). Calibration plot was produced to check the model calibration, which refers to closeness of the probability predicted by the developed model and the observed probability. Mean absolute error was calculated as the average difference between the predicted to the actual probability. Statistical analysis was performed using Stata 14 for windows, R software version 3.6.2 with MASS, rms, nomogramFormula, pROC, DynNom packages [[14], [15], [16], [17], [18]].

### 2.6 Human participant protection

The study protocol was approved by the Institutional Ethics Committee of ICMR-National Institute of Epidemiology, Chennai [NIE/IHEC/2016-04-01] and all the surveillance sites. Written informed consent was obtained from the parents of the suspected CRS patients before enrolling them in the surveillance.

## 3 Results

### 3.1 Descriptive epidemiology

Between November 2016 and December 2021, the 14 surveillance sites enrolled 3940 suspected CRS patients. Their mean age was 3.5 months (SD: 3.5) and 59.1% (n = 2329) were boys. About one-fifth (n = 813, 20.6%) were enrolled during newborn examination, while the majority were detected during their consultation with the sentinel health facility. Average age of mothers of suspected CRS patients was 25.9 (SD: 4.5) years (Table 1). About three fourth of the infants (n = 2843, 72.2%) were suspected of CRS due to structural heart defects, 579 (14.7%) had one or more of eye signs, 597 (15.2%) had hearing impairment, while mothers of 413 (10.5%) gave history of fever with rash when they were pregnant. Most (n = 3226, 81.9%) case-patients had one of the five criteria, 592 (15.0%) had two and 122 (3.1%) had more than 2 criteria for suspected CRS.

**Table 1:** Demographic and Clinical Characteristics of suspected CRS case-patients, 2016-21.

Demographic/Clinical details	Suspected CRS (n = 3940)	Lab confirmed CRS (n = 493)	Discarded cases (n = 3168)	P value
Age (months): mean (SD)	3.54 (3.54)	6.07 (4.20)	3.11 (3.14)	<0.001
Age of the mother (years): mean (SD)	25.94 (4.46)	26.04 (4.37)	25.95 (4.49)	0.6588
Gender				
Male	2,329 (59.11)	278 (56.39)	1,892 (59.72)	0.161
Female	1,611 (40.89)	215 (43.61)	1,276 (40.28)	
**ENT examination**				
Hearing impairment	399 (10.50)	131 (26.90)	244 (7.99)	<0.001
Not evaluated	141	6	113	
**Ophthalmic examination**				
Cataract	427 (11.01)	119 (24.29)	280 (8.97)	<0.001
Not evaluated	63	3	47	
Congenital glaucoma	51 (1.37)	11 (2.28)	36 (1.20)	0.058
Not evaluated	211	10	177	
Pigmentary retinopathy	61 (1.64)	27 (5.58)	30 (1.00)	<0.001
Not evaluated	213	9	180	
**Cardiovascular examination**				
Structural heart defect	2,877 (73.22)	382 (77.48)	2,312 (73.19)	0.044
Not evaluated	11	-	9	
**General examination**				
Splenomegaly	351 (8.92)	52 (10.55)	245 (7.74)	0.034
Not evaluated	3	-	3	
Purpura	43 (1.09)	13 (2.64)	24 (0.76)	0.001
Not evaluated	2	-	2	
Jaundice	413 (10.48)	40 (8.11)	304 (9.60)	0.294
**Central Nervous System examination**				
Microcephaly	1,904 (48.32)	249 (50.51)	1,501 (47.38)	0.196
Developmental delay	1,058 (27.54)	216 (44.72)	723 (23.40)	<0.001
Not evaluated	99	10	78	
Meningoencephalitis	93 (2.37)	9 (1.84)	68 (2.15)	0.651
Not evaluated	12	3	9	

P value based on compararision of proportions among lab confirmed and discarded cases

**Fig. 1:**
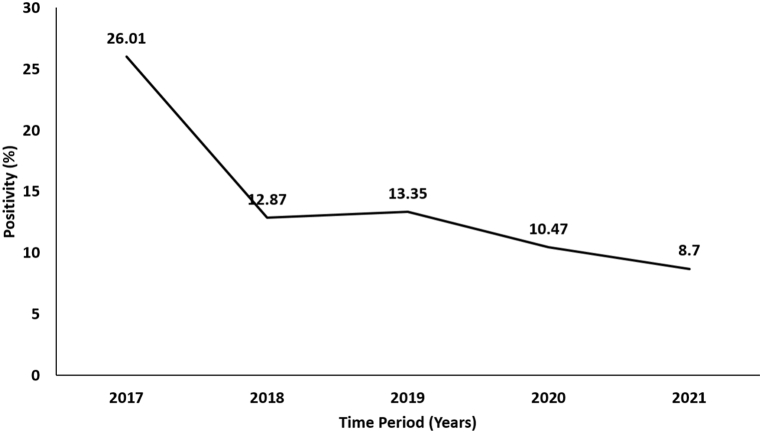
Trend graph of rubella positivity among suspected CRS case-patients, 2017-21.

Of the 3940 children suspected of CRS, 493 (12.5%) were categorized as laboratory confirmed, 71 (1.8%) as CRI, 208 (5.3%) as clinically compatible cases, and 3168 (80.4%) as discarded cases. The proportion of the laboratory confirmed CRS cases ranged between 3.7% and 23.2% (Supplementary Table 1) in different surveillance sites. The laboratory confirmed CRS cases were from 319 districts in the 21 Indian States and 4 union territories (Supplementary figure 2). The proportion of laboratory confirmed CRS declined from 26.0% in 2017 to 8.7% in 2021 (Fig. 1). In five sentinel sites, this proportion showed a significant decline (p < 0.001), whereas in six sites, the proportion of laboratory confirmed CRS was not different across the years. In one site (Bhubaneswar), the proportion increased from 15.9% in 2019 to 26.5% in 2021 (Supplementary Table 2). Of the 385 laboratory confirmed rubella patients tested for RT-PCR, 53 (13.8%) were positive.

### 3.2 Clinical details of laboratory confirmed CRS patients

Of the 493 laboratory confirmed CRS infants, 382 (77.5%) had structural heart defects; 178 (46.6%) had single defect and 204 (53.4%) had complex defects (Supplementary Table 3). One hundred and thirty-one (26.9%) patients had hearing impairment - 93 (71.0%) had bilateral defect, 12 (9.2%) had unilateral defect, information about laterality was not mentioned in 26 patients. In the ophthalmic examination, 119 (24.3%) had cataract, 27 (5.6%) had pigmentary retinopathy and eleven (2.3%) had congenital glaucoma. Other clinical features among the laboratory confirmed patients included microcephaly (n = 249, 50.5%), development delay (n = 216, 44.7%), splenomegaly (n = 52, 10.6%), jaundice (n = 40, 8.1%) within 24 h of birth, purpura (n = 13, 2.6%) and meningoencephalitis (n = 9, 1.8%) (Table 1). Of the 493 laboratory confirmed CRS infants, 103 died within 2 years of the diagnosis, with a case fatality of 20.9%.

### 3.3 Quality of surveillance

Of the 3940 infants with suspected CRS, 840 (21.1%) were born in sentinel surveillance health facilities, with the annual reporting rates ranging between 2.2 and 53.4 per 10,000 live births. Most suspected CRS patients (n = 3904, 99.1%) were adequately investigated in terms of collection of demographic characteristics, clinical examination for heart, eye and hearing (n = 3765, 95.6%), while adequate specimens were collected from 83.6% patients. Although, specimens were transported to the laboratories on the same day, 1656 (50.3%) sera were tested within 4 days. About a third (n = 179, 36.3%) laboratory confirmed CRS infants were detected within 3 months of birth. Of the 493 laboratory confirmed CRS patients, OP swabs were collected from 469 (95.1%), however, only 210 (53.8%) could be followed-up for viral excretion among eligible infants (n = 390) (Supplementary Table 4).

### 3.4 Comparison of clinical details of laboratory confirmed and discarded case-patients

We compared clinical details of 493 laboratory confirmed and 3168 discarded case-patients. In univariate analysis, compared to discarded case-patients, the laboratory confirmed CRS patients had higher odds (p < 0.05) of the following group A signs: pigmentary retinopathy (Odds ratio [OR]: 5.8, 95% confidence interval [CI]: 3.4–9.9), hearing impairment (OR: 4.2, 95% CI: 3.3–5.4), congenital cataract (OR: 3.3, 95% CI: 2.6–4.1), and structural heart defect (OR: 1.3, 95% CI: 1.0–1.6). The laboratory confirmed patients also had higher odds of purpura (OR: 3.6, 95% CI: 1.8–7.0), development delay (OR: 2.7, 95% CI: 2.2–3.2) and splenomegaly (OR: 1.4, 95% CI: 1.0–1.9) (Table 2).

**Table 2:** Comparison of group A and group B clinical signs among laboratory confirmed and discarded CRS case-patients.

	Univariate Model			Multivariable Model^a^		
Risk factors	Coefficients	OR (95% CI)	P value	Coefficients	AOR (95% CI)	P value
Group A signs						
Structural heart defect	0.23	1.26 (1.01,1.58)	0.044	2.30	3.82 (1.19, 12.18)	<0.001
Cataract	1.18	3.25 (2.56,4.14)	<0.001	2.05	7.75 (5.39, 11.15)	<0.001
Glaucoma	0.65	1.91 (0.97,3.78)	0.062	1.15	3.14 (1.21, 8.14)	0.018
Pigmentary Retinopathy	1.76	5.83 (3.43,9.89)	<0.001	1.90	6.70 (3.30, 13.59)	<0.001
Hearing impairment	1.44	4.24 (3.34,5.38)	<0.001	2.25	9.52 (5.61, 16.15)	<0.001
Structural heart defect* Hearing impairment				−0.96	3.80 (1.19, 12.15)	0.009
Group B signs						
Microcephaly	0.13	1.13 (0.94,1.37)	0.196			
Development delay	0.97	2.65 (2.17,3.23)	<0.001	0.33	1.39 (1.09, 1.77)	0.007
Purpura	1.27	3.55 (1.79,7.01)	<0.001	2.05	7.76 (3.21, 18.75)	<0.001
Meningo-encephalitis	−0.16	0.85 (0.42,1.72)	0.651			
Jaundice	−0.18	0.83 (0.59,1.17)	0.295			
Splenomegaly	0.34	1.41 (1.03,1.93)	0.035			

OR: Odds Ratio

AOR: Adjusted Odds Ratio

a Model adjusted for age in years.

Based on -2Log likelihood criterion, we selected all variables from univariate analysis into multivariable model except microcephaly, jaundice, meningo-encephalitis and splenomegaly. In the multivariable binary logistic regression model, the laboratory confirmed CRS patients were more likely to have hearing impairment (Adjusted OR [AOR]: 9.5, 95% CI: 5.6–16.2), cataract (AOR: 7.8, 95% CI: 5.4–11.2), pigmentary retinopathy (AOR: 6.7, 95% CI: 3.3–13.6), structural heart defect with hearing impairment (AOR: 3.8, 95% CI: 1.2, 12.2) and glaucoma (AOR: 3.1, 95% CI: 1.2, 8.1) from group A signs and purpura (AOR: 7.8, 95% CI: 3.2–18.8) and developmental delay (AOR: 1.4, 95% CI: 1.1–1.8) from group B signs (Table 2).

**Fig. 2:**
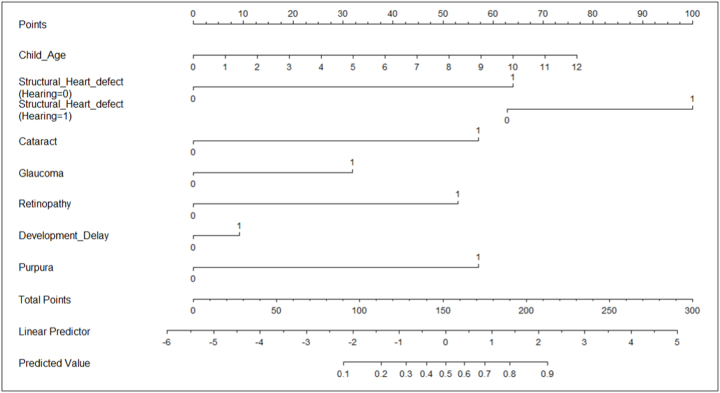
Nomogram for predicting the probability of CRS case-patients.

Based on the final risk model, the nomogram was developed, to find the risk probability of congenital rubella (Fig. 2). The model had good discrimination with an AUC of 0.81 (95% CI: 0.787–0.833) (Supplementary figure 3). The developed model was validated by bootstrapping with 1000 resamples and AUC after bootstrapping was 0.811. The optimism of bootstrap was 0.003 and bias corrected AUC was 0.807 indicating that the AUC after bootstrapping the risk model was almost similar to the AUC of the originally developed risk model. This indicated that the developed risk model was well discriminative of the laboratory confirmed and discarded cases.

Overall, the model appeared to be reasonably well calibrated based on the bias-corrected line near to ideal line (Supplementary figure 4). The apparent probability was almost closer to that of bias-corrected actual probability (mean absolute error: 0.019). An interactive nomogram based on developed risk model could be accessed at the following URL – https://icmr-nie.shinyapps.io/Rubella/(Supplementary figure 5).

## 4 Discussion

Our analysis of the surveillance data from 14 sentinel sites indicated that about 13% of the suspected CRS enrolled between 2016 and 2021 had laboratory evidence of rubella infection. One fifth of the laboratory confirmed patients died within 2 years of the diagnosis. The surveillance system met most of WHO quality indicators for surveillance, except monitoring for virus excretion, timeliness of detection of lab confirmed CRS infants and timeliness of laboratory reporting.

We were not able to precisely estimate the incidence of CRS cases using the surveillance data, as the population of the catchment area of the sentinel surveillance facilities was not precisely known. Moreover, not all suspected CRS patients residing in the catchment area would seek healthcare from the sentinel surveillance site. Hence, we considered the proportion of laboratory confirmed cases as an indicator to monitor the trend. Our analysis indicated that the proportion of laboratory confirmed cases declined from 26% in 2017 to 10.5% in 2020 and 8.7% in 2021. The declining trend could be on account of decreased transmission of rubella on account of increase in population immunity on account of MRCV SIAs. We observed a declining trend in test positivity in five sites, increasing trend in one site and no trend in the remaining six sentinel sites. However, our analysis of test positivity has one important limitation. The enrollment of suspected case-patients during 2020 and 2021 was lower, especially during the period when the lockdown was imposed in India following COVID-19 pandemic. It is therefore crucial to continue surveillance in coming years to document the progress made towards rubella control.

Early identification of laboratory confirmed CRS can help initiate corrective measures for various defect/s in children on account of CRS. Also, early identification of such babies could prevent further transmission in the community, by reducing the exposure of such infants to pregnant women especially in hospital settings, as infants with CRS are important source of transmission in the community [19]. The surveillance data indicated that laboratory confirmed cases were detected late, whereas the WHO surveillance quality indicator recommends that 80% of such infants should be identified within three months of birth [11,12]. Also, less than 20% of the suspected CRS patients were detected based on newborn examination whereas majority were enrolled during their consultation with the sentinel health facility. These findings point to the need for strengthening screening of newborn babies for birth defects suggestive for CRS, in India.

As India progresses towards the goal of elimination of rubella/CRS, there is a need to expand the CRS surveillance to identify most cases of CRS. In India, babies born in majority of the tertiary care hospitals in public sector are routinely screened for common birth defects. Further, the Rashtriya Bal Swasthya Karyakram (RBSK) launched in 2013 by the National Health Mission aims at early identification and intervention of children from birth to 18 years to cover 4 “Ds” – defects at birth, deficiencies, diseases, developmental delays including disability [20]. Under RBSK, newborns born at public health facilities are expected to be screened for eight birth defects (neural tube defect, Down syndrome, cleft lip/palate, clubfoot, developmental dysplasia of hip, congenital cataract, congenital deafness, congenital heart defects, and retinopathy of prematurity). The district early intervention centers (DEIC) established at the district hospitals are expected to play an important role in screening of newborns and provide referral support to tertiary care hospitals for further investigations and management [20,21]. The platform of screening newborn babies at tertiary care hospitals as well as districts hospitals under RBSK could be used for identifying the suspected CRS cases. Using the nomogram developed based on the CRS surveillance data, newborn with a prediction probability cutoff of 0.15 (sensitivity: 72%, specificity: 76%) identified from these facilities could be prioritized for serological investigations to confirm the rubella infection.

In conclusion, our analysis of the CRS surveillance data indicated rubella as a significant public health issue in India. The declining trend of test positivity among suspected CRS patients seen in the last two years needs to be monitored through continued surveillance in these sentinel sites. Further, as India approaches towards rubella elimination, existing platform of birth defect surveillance could be used to expand CRS surveillance.

## Author contribution statement

Manoj Murhekar; Devika Shanmugasundaram: Conceived and designed the experiments; Analyzed and interpreted the data; Wrote the paper.

Gajanan Sapkal: Conceived and designed the experiments; Performed the experiments.

Nivedita Gupta: Conceived and designed the experiments

Sanjay Verma; Kuldeep Singh; Bhagirathi Dwibedi; Shally Awasthi; S. Mahantesh; Himabindu Singh; Sridhar Santhanam; Nivedita Mondal; Geetha S; Priya Sreenivasan; Shikha Malik; Manish Jain; Rajlakshmi Viswanathan; Shalini Tripathi; Bhupeshwari Patel; R. Sabarinathan; Mini P. Singh; R. K. Ratho; Vijaylakshmi Nag; Ravishekhar Gadepalli; Tapas Kumar Som; Baijayantimala Mishra; Amita Jain; M. Ashok; Devara Sudha Madhuri; V Sudha Rani; Asha Mary Abraham; Deepa John; Rahul Dhodapkar; Syed Ali. A; Debasis Biswas; Deepashri Pratyeke; Ashish Bavdekar; Jayant Prakash; Varsha Singh; Nidhi Prasad; Jaydeb Ray; Agniva Majumdar; Shanta Dutta: Performed the experiments.

Akhil Sharma; Aniruddha Ghosh; Arun Alexander; Arun Baranwal; Avinash Anantharaj; Adhisivam Bethou; Dolat S. Shekhawat; G. Kiruthika; Jagat Ram; Madhu Gupta; Mamatha Gowda; Manoj K Rohit; Nabaneeta Dash; Naveen Sankhyan; Nidhi Kaushal; Niranjan Hunasanahalli Shivanna; Nirupama Kasturi; P. Prem Kumar; Parul Chawla Gupta; Pradeep Kumar Gunasekaran; Pratibha Singh; Praveen Kumar; Sanjay Kumar Munjal; Siddharth Agarwal; Suhani Manasa; Suruchi Shukla; Urvashi Nehra; Valsan P Verghese; Varuna Vyas; Vikas Gupta: Contributed reagents, materials, analysis tools or data.

## Data availability statement

Data included in article/supplementary material/referenced in article.

## Declaration of competing interest

The authors declare that they have no known competing financial interests or personal relationships that could have appeared to influence the work reported in this paper.
