# Supplementary material for: Congenital rubella syndrome surveillance in India, 2016–21: Analysis of five years surveillance data
Source: Heliyon. 2023 May 9;9(5):e15965. doi: 10.1016/j.heliyon.2023.e15965 (PMC10209330; doi:10.1016/j.heliyon.2023.e15965)
Supplement: Multimedia component 1 [file mmc1.docx]

**Supplementary material**

**Supplementary Figure 1: Congenital Rubella Syndrome Surveillance network in India**

**
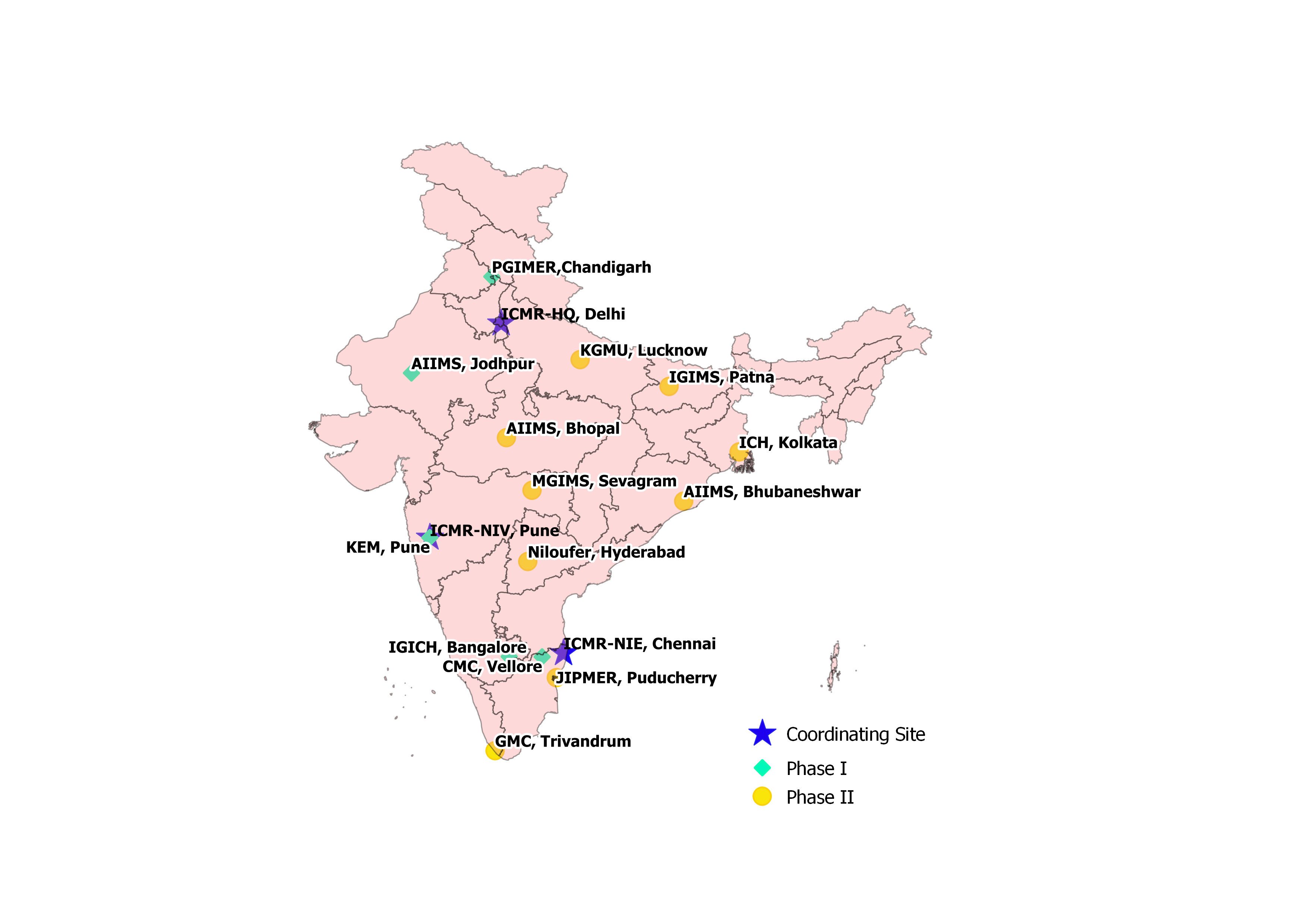
**

**Supplementary Figure 2: Spot map of laboratory confirmed CRS cases, 2016-21**

**
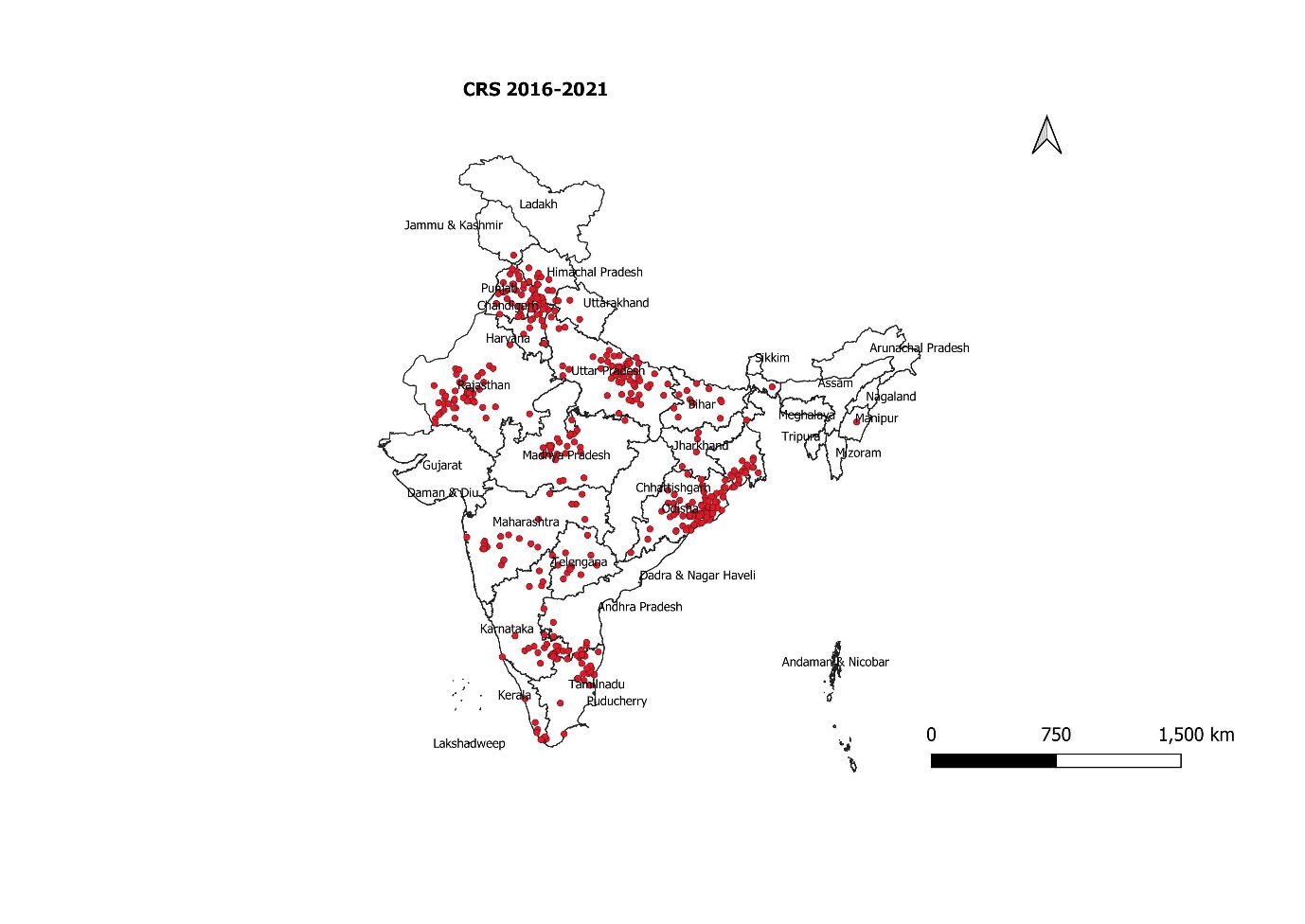
**

**Supplementary Figure 3: Receiver Operating characteristic (ROC) curve showing the performance of the risk model in identifying CRS**


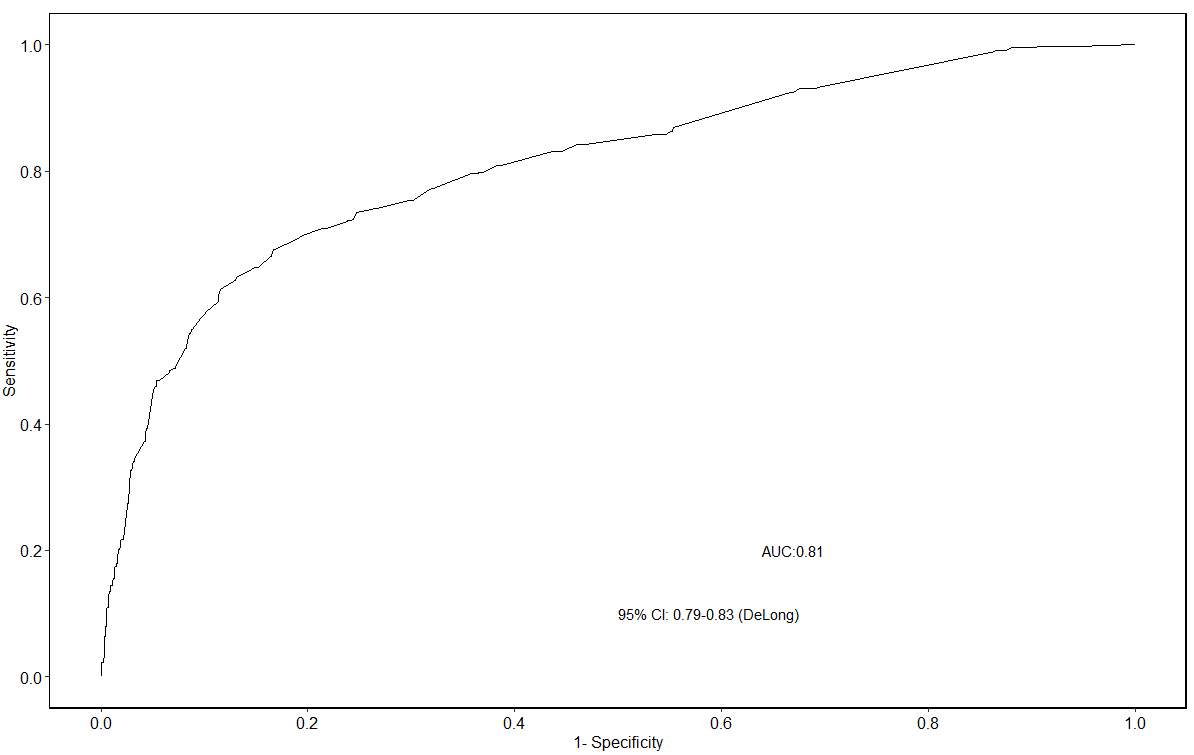


**Supplementary Figure 4: Graphical representation of the apparent probability against the bias corrected actual probability based on 1000 bootstrap replicates for assessing the developed multivariable binomial logistic regression model**


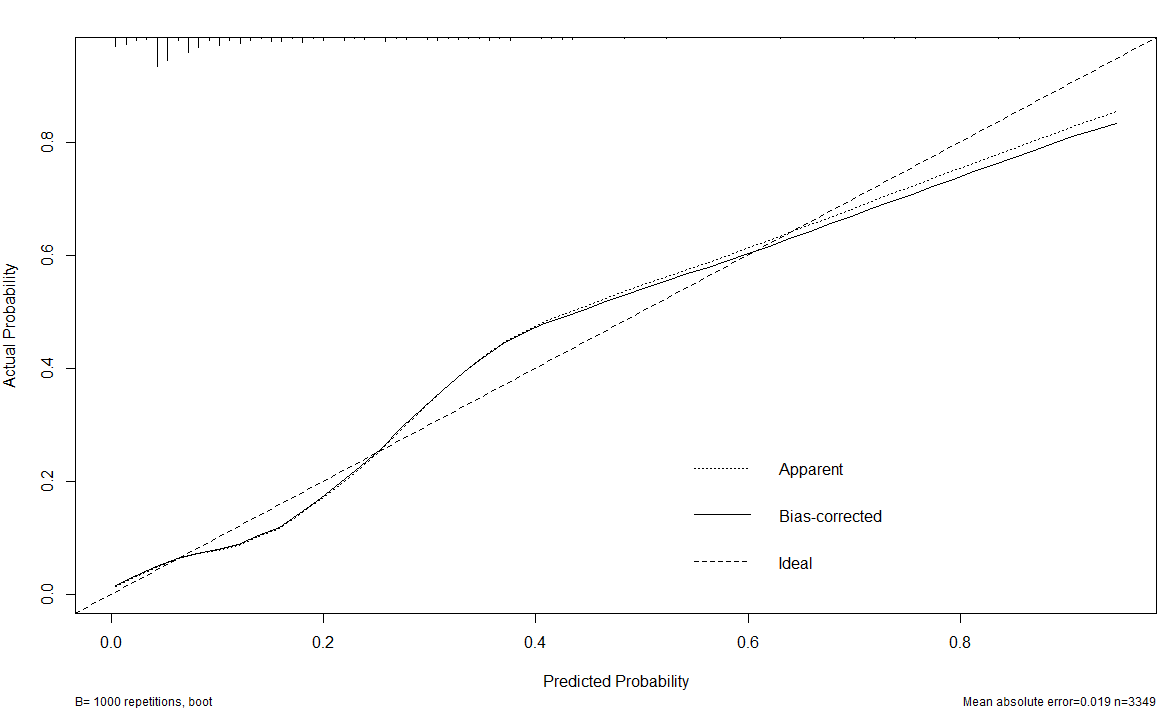


**Supplementary Figure 5: Dynamic nomogram of the logistic regression model**


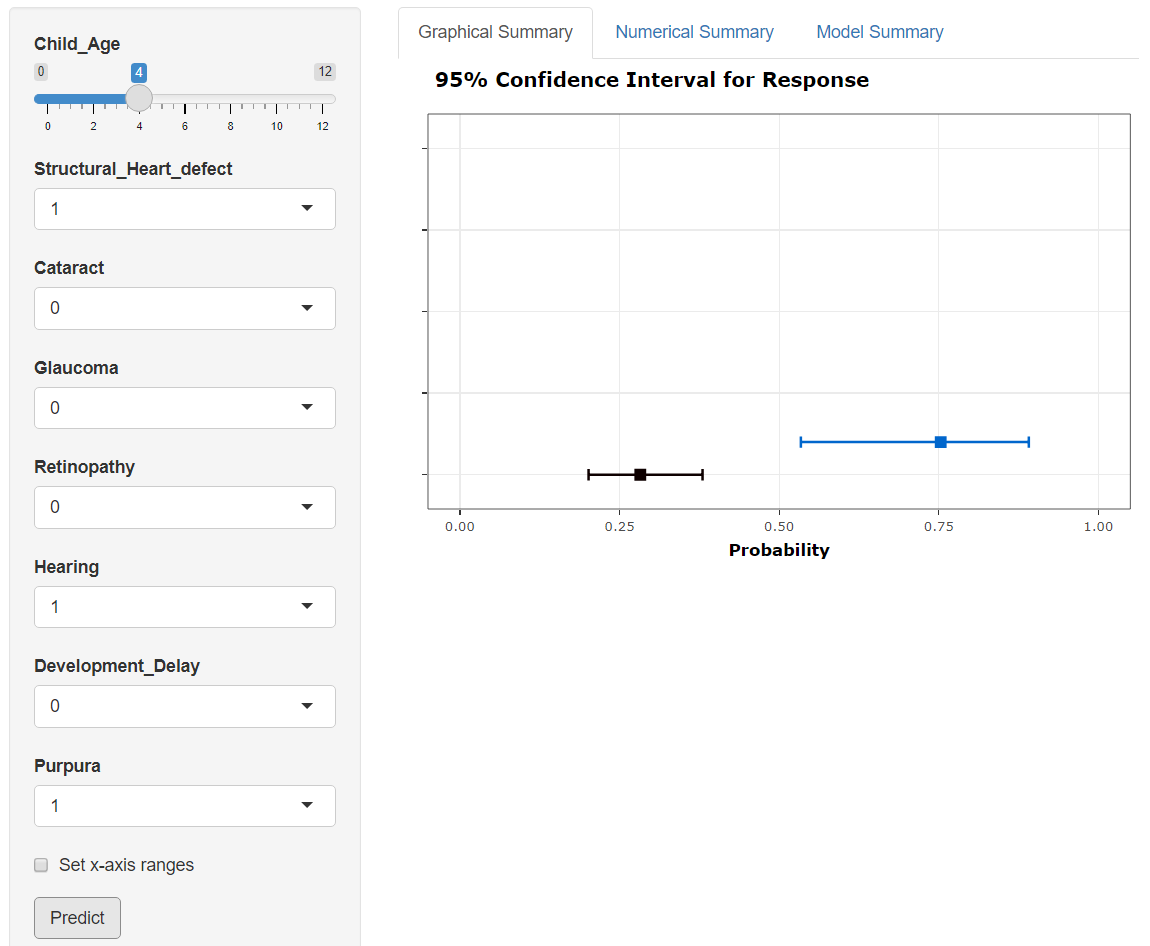

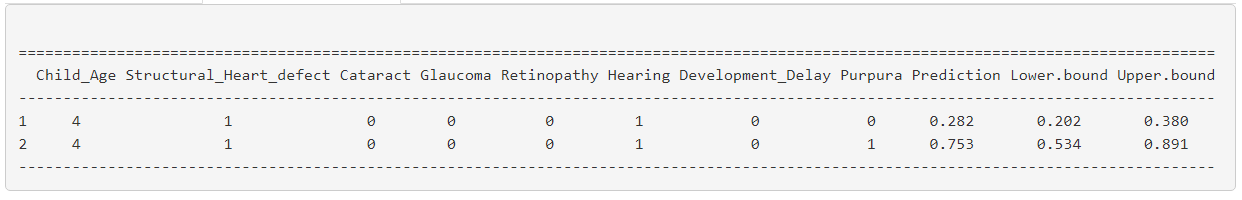


**Supplementary Table 1: Suspected and laboratory confirmed CRS patients by surveillance site, 2016-21**

| **Sentinel Sites** | **Enrolled** | **Number Positive (%)** |
| --- | --- | --- |
| All India Institute of Medical Sciences (AIIMS), Jodhpur | 544 | 64 (11.8) |
| Christian Medical College (CMC), Vellore | 287 | 17 (5.9) |
| Indira Gandhi Institute of Child Health (IGICH), Bangalore | 308 | 49 (15.9) |
| King Edward Memorial Hospital (KEM), Pune | 157 | 25 (15.9) |
| Post Graduate Institute of Medical Education and Research (PGIMER), Chandigarh | 673 | 89 (13.2) |
| All India Institute of Medical Sciences (AIIMS), Bhubaneshwar | 475 | 110 (23.2) |
| Institute Of Child Health (ICH), Kolkatta | 54 | 6 (11.1) |
| Niloufer Hospital, Hyderabad | 301 | 11 (3.7) |
| All India Institute of Medical Sciences (AIIMS), Bhopal | 155 | 27 (17.4) |
| Mahatma Gandhi Institute of Medical Sciences (MGIMS), Sewagram | 107 | 5 (4.7) |
| King George’s Medical University (KGMU), Lucknow | 434 | 57 (13.1) |
| Government Medical College (GMC), Trivandrum | 201 | 11 (5.5) |
| Indira Gandhi Institute of Medical Sciences (IGIMS), Patna | 64 | 7 (10.9) |
| Jawaharlal Institute of Postgraduate Medical Education and Research (JIPMER), Pondicherry | 180 | 15 (8.3) |
| Overall | 3940 | 493 (12.5) |

**Supplementary Table 2: Laboratory confirmed rubella positivity in different surveillance site by year, 2017-21**

| **Sentinel Sites** | **2017** | | **2018** | | **2019** | | **2020** | | **2021** | | **P value^*^** |
| --- | --- | --- | --- | --- | --- | --- | --- | --- | --- | --- | --- |
|  | **Enrolled** | **n (%)** | **Enrolled** | **n (%)** | **Enrolled** | **n (%)** | **Enrolled** | **n (%)** | **Enrolled** | **n (%)** |  |
| AIIMS, Jodhpur | 61 | 18 (29.5) | 98 | 11 (11.2) | 190 | 19 (10.0) | 105 | 8 (7.6) | 81 | 4 (4.9) | <0.001 |
| CMC, Vellore | 39 | 7 (17.9) | 28 | 3 (10.7) | 70 | 5 (7.1) | 61 | 0 | 87 | 0 | <0.001 |
| IGICH, Bangalore | 46 | 13 (28.3) | 37 | 9 (24.3) | 60 | 12 (20.0) | 50 | 5 (10.0) | 105 | 5 (4.8) | <0.001 |
| KEM, Pune | 55 | 5 (9.1) | 55 | 11 (20.0) | 38 | 9 (23.7) | 9 | 0 |  |  | 0.3554 |
| PGIMER, Chandigarh | 72 | 28 (38.9) | 116 | 9 (7.8) | 197 | 23 (11.7) | 124 | 8 (6.4) | 147 | 9 (6.1) | <0.001 |
| AIIMS, Bhubaneshwar |  |  |  |  | 132 | 21 (15.9) | 147 | 37 (25.2) | 196 | 52 (26.5) | 0.0319 |
| ICH, Kolkatta |  |  |  |  | 30 | 5 (16.7) | 11 | 1 (9.1) | 13 | 0 | 0.1067 |
| Niloufer, Hyderabad |  |  |  |  | 101 | 5 (4.9) | 98 | 2 (2.0) | 102 | 4 (3.9) | 0.6982 |
| AIIMS, Bhopal |  |  |  |  | 44 | 8 (18.2) | 37 | 12 (32.4) | 74 | 7 (9.5) | 0.1212 |
| MGIMS, Sewagram |  |  |  |  | 28 | 2 (7.1) | 43 | 1 (2.3) | 36 | 2 (5.6) | 0.8240 |
| KGMU, Lucknow |  |  |  |  | 212 | 41 (19.3) | 109 | 9 (8.3) | 113 | 7 (6.2) | <0.001 |
| GMC, Trivandrum |  |  |  |  | 29 | 1 (3.4) | 78 | 8 (10.3) | 94 | 2 (2.1) | 0.2656 |
| IGIMS, Patna |  |  |  |  |  |  | 25 | 4 (16.0) | 39 | 3 (7.7) | - |
| JIPMER, Pondicherry |  |  |  |  |  |  | 49 | 4 (8.2) | 131 | 11 (8.4) | - |
| Overall | 273 | 71 (26.0) | 334 | 43 (12.9) | 1,131 | 151 (13.3) | 946 | 99 (10.5) | 1,218 | 106 (8.7) | <0.001 |

* χ^2^ test for trend

**Supplementary Table 3: Characteristics of structural heart defects among laboratory confirmed congenital rubella syndrome cases — Congenital Rubella Sentinel Surveillance, India, November 2016– December 2021**

| **Type of defects*** | **Laboratory-confirmed CRS with structural heart defects (n = 382)** |
| --- | --- |
| **Single cardiac defects** |  |
| PDA | 60 (33.7) |
| VSD | 48 (27.0) |
| ASD/PFO | 41 (23.0) |
| PH/PAH | 3 (1.7) |
| PS | 2 (1.1) |
| Other | 24 (13.5) |
| **Total** | **178 (46.6)** |
| **Complex cardiac defects** | **204 (53.4)** |

Abbreviation: CRS = congenital rubella syndrome; PDA = patent ductus arteriosus; ASD = atrial septal defect; PFO = patent foramen ovale; VSD = ventricular septal defect; PS = pulmonary stenosis;

*Classified as simple or complex defects according to the NIH National Heart, Lung, and Blood Institute definition of Types of Congenital Heart Defects. Available at: <https://www.nhlbi.nih.gov/health-topics/congenital-heart-defects>

**Supplementary Table 4: Surveillance quality indicators**

| **Criteria** | **Threshold** | % |
| --- | --- | --- |
| **1. Reporting rate**  (National annual rate of suspected CRS cases) | ≥1/10,000 LB | 2.2 – 53.4  per 10,000 LB |
| **2. Adequacy of investigation** |  |  |
| (a) % suspected CRS with key data points | >80% | 99.1  (3904/3940) |
| (b) % suspected CRS patients who underwent clinical evaluation for heart  defects, eye signs and hearing. |  | 95.6 (3765/3940) |
| **3. Specimen collection/testing adequacy**  (% suspected cases with adequate specimen collected+ tested) | ≥80% | 83.6  (3293/3940) |
| **4. Adequacy of specimens for viral detection**  (% confirmed cases with adequate specimens for virus detection) | ≥80% | 95.1  (469/493) |
| **5. Monitoring for virus excretion**  (% confirmed CRS cases followed up for viral excretion) | ≥80% | 53.8  (210/390) |
| **6. Timeliness of detection**  (% confirmed CRS cases detected within 3 months of birth) | ≥80% | 36.3  (179/493) |
| **7. Timeliness of specimen transport**  (Proportion of specimens received at lab within 5 days of collection) | ≥80% | 100.0 |
| **8. Timeliness of reporting laboratory results**  (% test results reported within 4 days of receipt of specimen) | ≥80% | 50.3  (1656/3293) |
